# The Ability of Neonatal Mice to Develop Immunity to *Mycobacterium tuberculosis* Shows Sex Differences, with Females Displaying Evidence of an Enhanced Immune Response

**DOI:** 10.33696/immunology.7.225

**Published:** 2025

**Authors:** Mrinal K. Ghosh, Ameae M. Walker

**Affiliations:** 1 Division of Biomedical Sciences, School of Medicine, University of California, Riverside, CA 92521, USA

**Keywords:** Improvement in neonatal immunity, Sex differences in neonatal immunity, Tuberculosis, Bi-functional CD8^+^ T cells, Perinatal testosterone surge, Male bias in TB susceptibility

## Abstract

Using four core genotypes (FCG) mice, we have previously shown a larger number of CD4^+^ and CD8^+^ T cells in the spleens of female mice, a sex difference that develops by postnatal day 7 and is retained through adulthood. This difference in splenic T cell number is a consequence of reduced thymic egress and reduced splenic seeding in male mice, caused in part by the male-specific perinatal surge of testosterone, and in part by *Sry*, which is overexpressed in this model. Here, we used the background strain for FCG mice (C57BL/6J) to ask whether sex influenced actual immunity in the postnatal period. Pups were immunized on postpartum days 1 or 3 with *Mycobacterium tuberculosis* (Mtb), challenged on day 7 with Mtb purified protein derivative (PPD), and sacrificed on day 8. Subsequent *ex vivo* challenges of splenocytes showed PPD-stimulated CD8^+^ responses (increased CD8^+^, increased CD8^+^CD44^hi^, decreased CD8^+^CD44^hi^CD127^−/lo^) but no differences between males and females. However, when CD8^+^ T cells were analyzed for IFN-γ and IL-2 production, although there was no sex difference in mono-functional IFN-γ^+^ (100%) or IL-2^+^ (67%), only females (0% of males and 42% of females) produced bi-functional (IFN-γ^+^IL-2^+^) cells. *Ex vivo* PPD-stimulated responses of other relevant cells from the spleen showed no sex differences in dendritic cells (CD11c^+^CD86^+^IL-6^+^) but females had more (3-fold) IL-6-producing macrophages (F4/80^+^CD86^+^IL-6^+^) and reduced T regulatory cells (CD4^+^CD25^+^Foxp3^+^). We conclude that some sex differences in immunity are evident at one week of age in Mtb immunized mouse pups, with females exhibiting qualitatively superior Mtb-specific immune responses.

## Introduction

The World Health Organization reported 10.8 million new cases of tuberculosis (TB) in 2023, with 11% occurring in children (0–14 years old). In fact, in 2023 after the COVID pandemic, TB returned to being the world’s leading cause of death from a single infectious agent, with children accounting for 15% of total deaths [1]. There are only limited treatment options for TB in children, who, in addition to succumbing to the disease themselves, can act as a reservoir for future cases of TB in adults. Thus, research with the goal of improving effective vaccination in neonates and infants is of the utmost importance [2]. Of significance in the world health organization epidemiological data [1] is the fact that males are more likely to become infected with *Mycobacterium tuberculosis* (Mtb), even in the 0–4-year-old subgroup.

The Bacillus Calmette–Guérin vaccine (BCG) is currently the only licensed vaccine for TB. After BCG vaccination, Vekemans *et al*. [3] found Gambian infants mounted a Th1 immune response to mycobacterial antigens. BCG vaccinated young mice also exhibit a Th1 response [4,5]. There remains, however, insufficient understanding of the development of immunity to *Mycobacterium tuberculosis* (Mtb) within the neonatal period, including whether there is a sex difference, as the human epidemiological data would suggest.

Using the four core genotypes (FCG) mouse model, in which contributions to sex differences by chromosomal content can be separated from those attributable to gonadal secretions, we have previously shown that there are sex differences in the number and percentages of CD4^+^ and CD8^+^ T cells within the spleens of male and female pups. Females have about twice the number of CD4^+^ T cells and about seven-fold the number of CD8^+^ T cells. These sex differences become evident by postnatal day 7 and are maintained through adulthood. Experimental manipulation demonstrated that reduced numbers of T cells in the male spleen were a consequence of both reduced thymic egress and reduced splenic seeding, caused in part by the male-specific perinatal surge of testosterone, and in part by *Sry* [6]. Since the sex difference in CD8^+^ T cell number was largely due to an indirect effect of *Sry* expression [6], and *Sry* is overexpressed in male FCG mice [7], it was important to analyze sex differences in CD8^+^ T cell-mediated neonatal immunity in the background strain, C57BL/6J. C57BL/6J mice are Th1 biased, have been used previously to examine some aspects of neonatal immunity, and have served as a preclinical model for TB vaccine studies [4,5,8,9]. However, no previous study to our knowledge has examined the effect of sex on the neonatal development of immunity following BCG vaccination. Male humans as well as mice have a perinatal surge in testosterone [10], thereby making our study of the neonatal period in a mouse model relevant to the human situation.

Because our analysis of immunity was during the nursing period when pups are newly exposed to gram negative bacteria from several sources, including maternal suckling [11], we also separately examined the impact of adding lipopolysaccharide (LPS) to the immunization protocol. LPS is an integral component of the outer membrane of Gram-negative bacteria, and has the capacity to activate dendritic cells [12], which may affect the development of immunity. There also exist multiple pathways for transduction of an LPS signal in macrophages [13].

The results show sex differences in the development of immunity to Mtb that are consistent with immunization producing better protection from TB in females.

## Materials and Methods

### Mice

C57BL/6J mice were purchased from The Jackson Laboratory (Bar Harbor, ME). Animal use for this project was approved by the Institutional Animal Care and Use Committee of the University of California, Riverside.

### Immunization

Newborn day 1 pups were immunized by intraperitoneal (i.p.) injection of heat-killed Mtb (15 μg in 20 μL of Dulbecco’s PBS). On day 7, pups were challenged i.p. with 5 μl (0.25 United States tuberculin units) of tuberculin purified protein derivative (PPD). In a separate protocol, day 3 pups were immunized by i.p. injection of heat-killed Mtb (15 μg in 20 μl of Dulbecco’s phosphate-buffered saline (Dulbecco’s PBS) along with 20 μl of lipopolysaccharide (LPS) (*Escherichia coli* O111:B4, Sigma-Aldrich, MO) (0.5 μg/ml in Dulbecco’s PBS). On day 7, pups were challenged with 5 μl PPD (0.25 United States tuberculin units) for 24 h before sacrifice.

### Splenocyte stimulation

Freshly isolated (within 1 h) splenocytes with >95% viability were incubated at 1 × 10^6^ cells/ml in RPMI 1640 supplemented with 10% fetal bovine serum (FBS; Life Technologies, Grand Island, NY) in the presence of purified anti-CD28 antibody (37.51; 1 μg/ml) (BD Biosciences) in the presence or absence of PPD (0.25 tuberculin units/ml). After 1 h, BD GolgiStop was added (final concentration of 2.0 mM) for an additional 16–18 h before harvesting. Cells were then stained for surface antigens by incubation in the appropriate antibody or antibodies (co-incubated if more than one) diluted in Dulbecco’s PBS plus 0.5% bovine serum albumin (BSA;30 min), washed 3 times with Dulbecco’s PBS containing 0.5% (BSA), permeabilized and fixed and washed using BD Biosciences Cytofix/Cytoperm (San Jose, CA) reagents for 20 min, stained for intracellular molecules (co-incubated if more than one) for 1 h followed by a second series of washes in BD reagent before final fixation in 2% paraformaldehyde in Dulbecco’s PBS. All steps were at 4°C. For this functional assay, a response was considered positive when the degree was at least twice that of the control (-PPD) value. Purified anti-CD3 antibody was used as a positive control. No data were excluded from analysis.

### Flow cytometry

All reagents and antibodies were purchased from eBioscience (San Diego, CA) or BD Biosciences: anti-CD4 (RM4–5), anti-CD8 (53–6.7), anti-CD11c (HL3), anti-CD44 (IM7), anti-F4/80 (BM8), anti–IFN-γ (XMG1.2), anti–IL-2 (JES6–5H4), and mouse IgG2a and rat IgG2b isotype controls. Fc receptors were blocked using purified rat anti-mouse CD16/CD32 (2.4G2) (0.5 μg/100 μl). Flow cytometry acquired viable cells, which were further gated on the basis of forward *versus* side scatter for single cells and then analyzed for staining with conjugated antibodies. For conjugated antibodies, fluorochrome conjugates were used to stain BD Biosciences CompBeads to set the equipment for compensation controls along with non-stained controls. In each analysis, appropriate isotype controls were used to define gate settings. Stained cells were acquired using the same gate settings as for isotype and fluorochrome stained CompBeads. Stained cells were acquired using a FACSAria (BD Biosciences), and analyzed by FlowJo software, version 9 (Tree Star, Ashland, OR). No data were excluded from analysis. Representative scatter plots are shown in Supplementary Figures 1–5.

### Statistical analysis

Statistical significance was determined using Student’s t test (two-tailed), with Bonferroni corrections, where appropriate. Data are expressed as means ± SD, and p<0.05 was considered statistically significant. As mentioned above, no data were excluded from the analyses.

## Results

### General CD8^+^ cell responses

**Figure 1.**
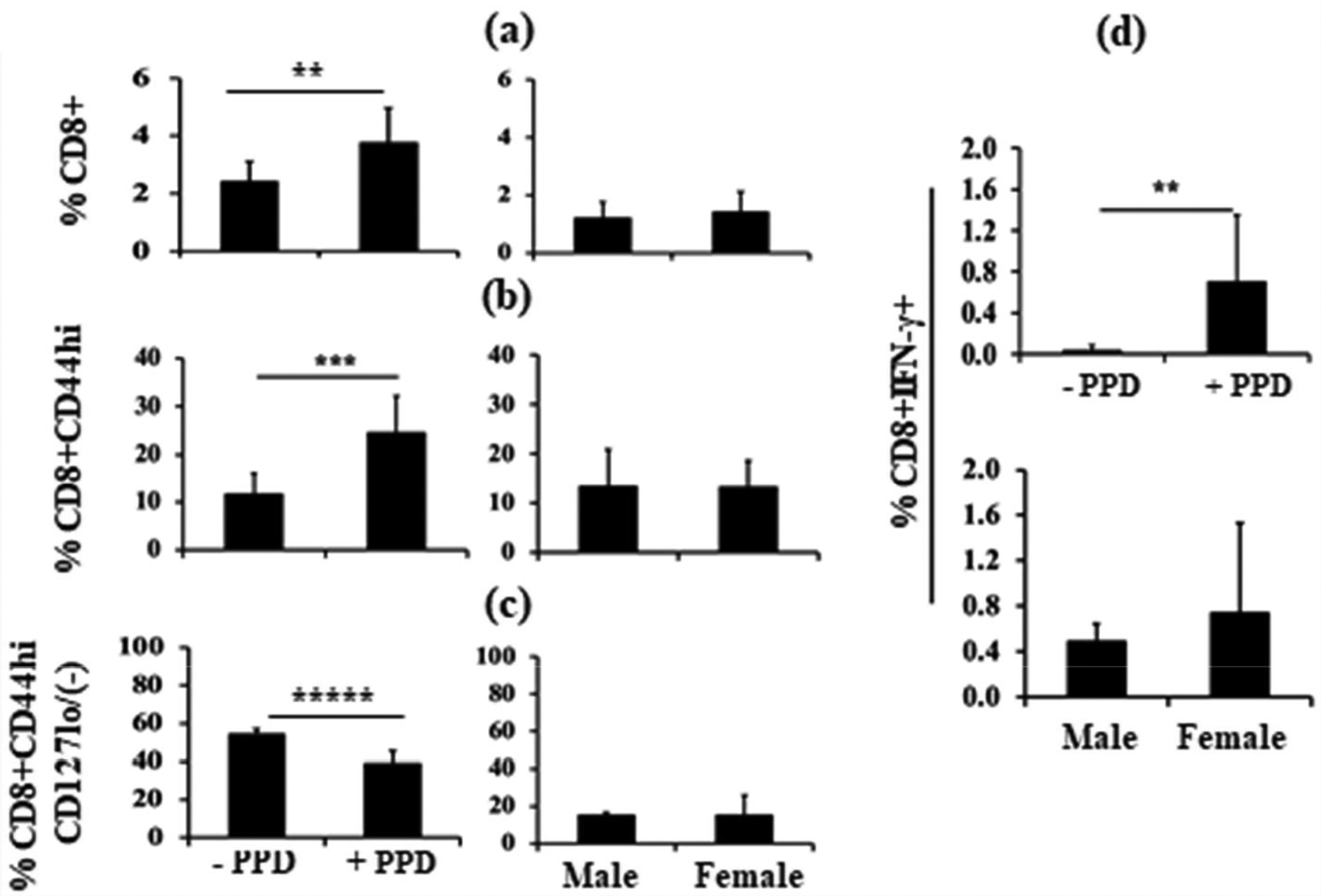
General CD8+ T Cell Responses. Day1 pups (n=10) were immunized with intraperitoneal Mtb and challenged with PPD on day 7 for 24 h before sacrifice. Isolated splenocytes were then stimulated *ex vivo* with or without PPD for 16 – 18 h. Expansion of CD8^+^ T cells upon PPD stimulation is shown (**1a**, left panel), and no sex (male, n=3; Female, (n=7) differences were evident (**1a**, right panel). Similarly, CD8^+^CD44hi T cells also expanded after PPD stimulation (**1b**, left panel) and again there was no significant sex difference (**1b**, right panel). Figure 1c shows CD8^+^CD44hiCD127(−/lo) T cells after *ex vivo* PPD stimulation; this cell population contracted significantly (left panel), with no sex differences (right panel). Figure 1d shows IFN-γ-producing CD8^+^ T cells after *ex vivo* PPD stimulation as compared to control. PPD stimulation significantly increased IFN-γ production by CD8^+^ T cells (upper panel), but there was no significant difference in %CD8^+^ IFN-γ^+^ T cells between males and females after control subtraction (bottom panel). Data are expressed as means ± SD, and *p<0.05 was considered statistically significant. **p<0.01, ***p<0.001, *****p<0.00001.

Mouse pups were immunized with Mtb on day 1 and challenged with PPD on day 7 for 24 h. Pup splenocytes were then stimulated with PPD *ex vivo* overnight. Figure 1a shows an expected expansion of CD8^+^ T cells upon antigen stimulation (left panel, +PPD), but no significant difference between male and female pups (right panel, which are both +PPD with -PPD subtracted). Similarly, CD8^+^CD44^hi^ activated populations significantly increased after PPD stimulation (Figure 1b, left panel), with no sex difference (right panel). Conversely, Figure 1c shows a highly significant CD8^+^CD44^hi^CD127(-/lo) T cell population contraction after PPD stimulation (left panel), but again no sex differences are evident (right panel). Finally for this figure, panel d shows an increase in the percentage of CD8^+^ T cells secreting IFN-γ in response to *ex vivo* stimulation with PPD (upper panel). Consistent with the other measures, however, male *versus* female responses were not significantly different (lower panel).

### Phenotypic characterization of PPD-specific CD8^+^ IFN-γ^+^ T cells

**Figure 2.**
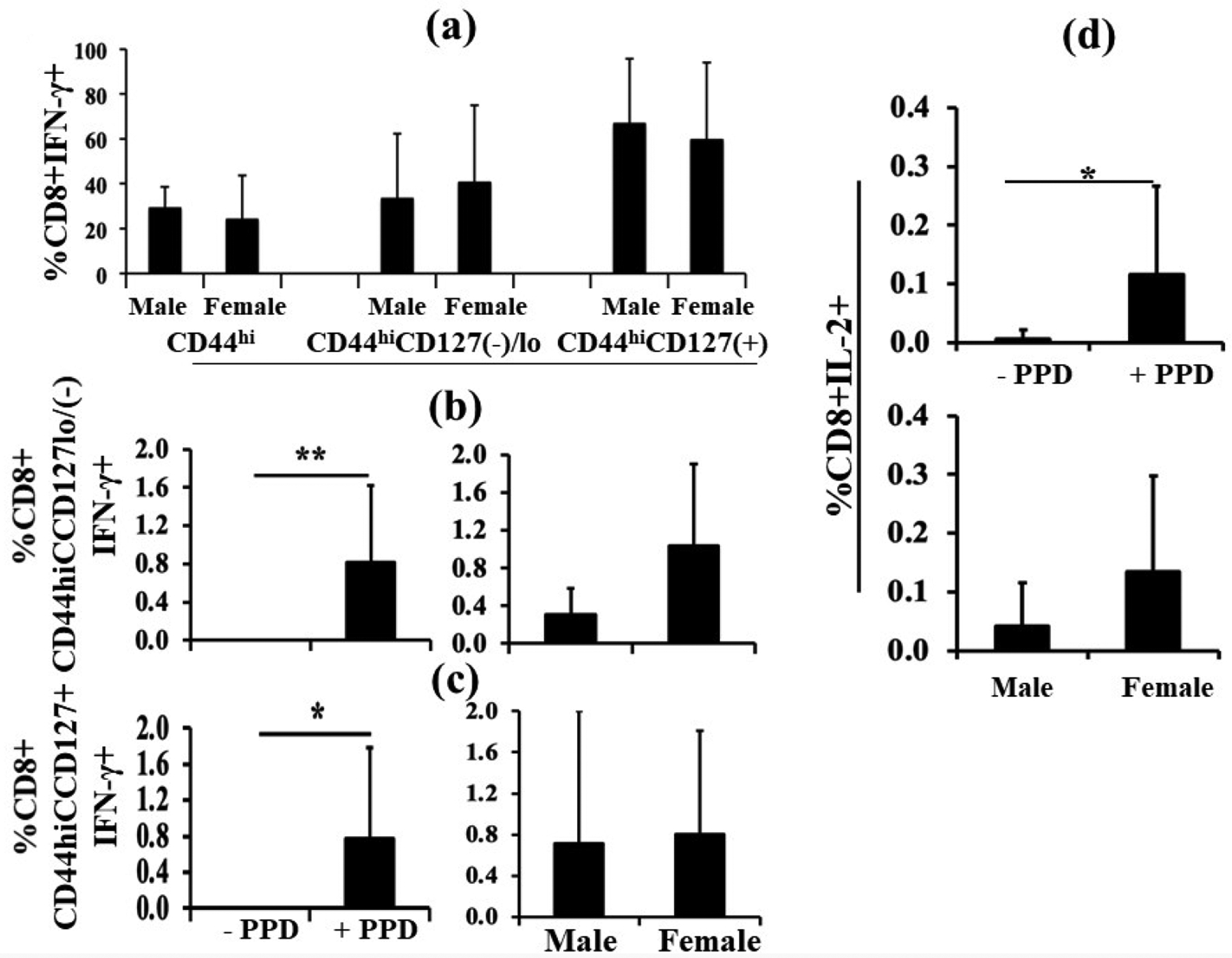
Phenotypic characterization of PPD-specific CD8^+^ IFN-γ^+^ T cells. Using the same *in vivo* treatments as for Figure 1, Figure 2a shows the phenotypes: CD44^hi^, CD44^hi^CD17(−/lo), and CD44^hi^CD127^+^ of CD8^+^ IFN-γ-producing T cells after *ex vivo* PPD stimulation (n=10). There were no significant differences between males (n=3) and females (n=7). PPD-specific IFN-γ production by CD8^+^CD44^hi^CD127(−/lo) T cells (**2b**, left panel) and negative control-subtracted male *versus* female data were not significantly different (**2b**, right panel), although there was a trend suggesting a greater response in female neonates. Figure 2c shows the percentage of PPD-specific IFN-γ production by CD8^+^CD44^hi^CD127^+^ T cells (**2c**, left panel). There was no male *versus* female difference (**2c**, right panel). Figure 2d shows IL-2 production after PPD stimulation by CD8^+^ T cells (**2d**, upper panel). The control-subtracted values were not significantly different between male and female pups (**2d**, lower panel). Data are expressed as means ± SD, and *p<0.05 was considered statistically significant. *p<0.05, **p<0.01.

Because the background rate of proliferation of memory-phenotype cells is increased following exposure to infectious agents, and is controlled by interferons, IFN-γ-secreting CD8^+^ T cells were further characterized for memory phenotype (CD44^hi^). CD44^hi^ T cells represent the long-lived progeny of T cells responding to various antigens [14]. Analysis revealed about 30% of CD8^+^IFN-γ^+^ cells in the pup spleens were CD44^hi^. However, there was no difference between males and females (Figure 2a, left).

CD127 (interleukin-7 receptor α chain) plays a critical role in the dynamic regulation of memory T cells. About 40% of the CD8^+^ IFN-γ^+^CD44^hi^ T cells were CD127 negative (−)/lo (Figure 2a, middle), meaning that the cells are likely to be effector or effector memory cells. Again, no sex differences were evident. The remaining cells were positive for CD127 expression (Figure 2a, right). Antigen-specific IFN-γ-producing CD8^+^ T cells expressing CD127 likely belong to the central memory cell category [15]. Once again, male *versus* female comparisons revealed no significant difference (Figure 2a, right).

When gating on the CD8^+^CD44^hi^ population further categorized as CD127^−/lo^ or CD127^+^, one can see CD8^+^CD44^hi^ CD127^−/lo^ T cells responded well to PPD stimulation by secreting IFN-γ (Figure 2b, left panel) but there was no significant difference between males and females. However, the magnitude of the response (mean) in females was more than twice that in males (right panel). In the CD8^+^CD44^hi^CD127^+^ T cell population, there was a significant response to PPD (Figure 2c, left panel) and again no significant difference in IFN-γ secretion between males and females in the +PPD samples (Figure 2c, right panel). In this case, the mean response to PPD in males and females was very similar.

PPD stimulation also resulted in a significant increase in IL-2 production by splenic CD8^+^ T cells (Figure 2d, upper panel). Although there was no significant sex difference, once again the mean magnitude of the response in females was more than twice that in males (Figure 2d, lower panel).

### Adding LPS to the immunization protocol

**Figure 3.**
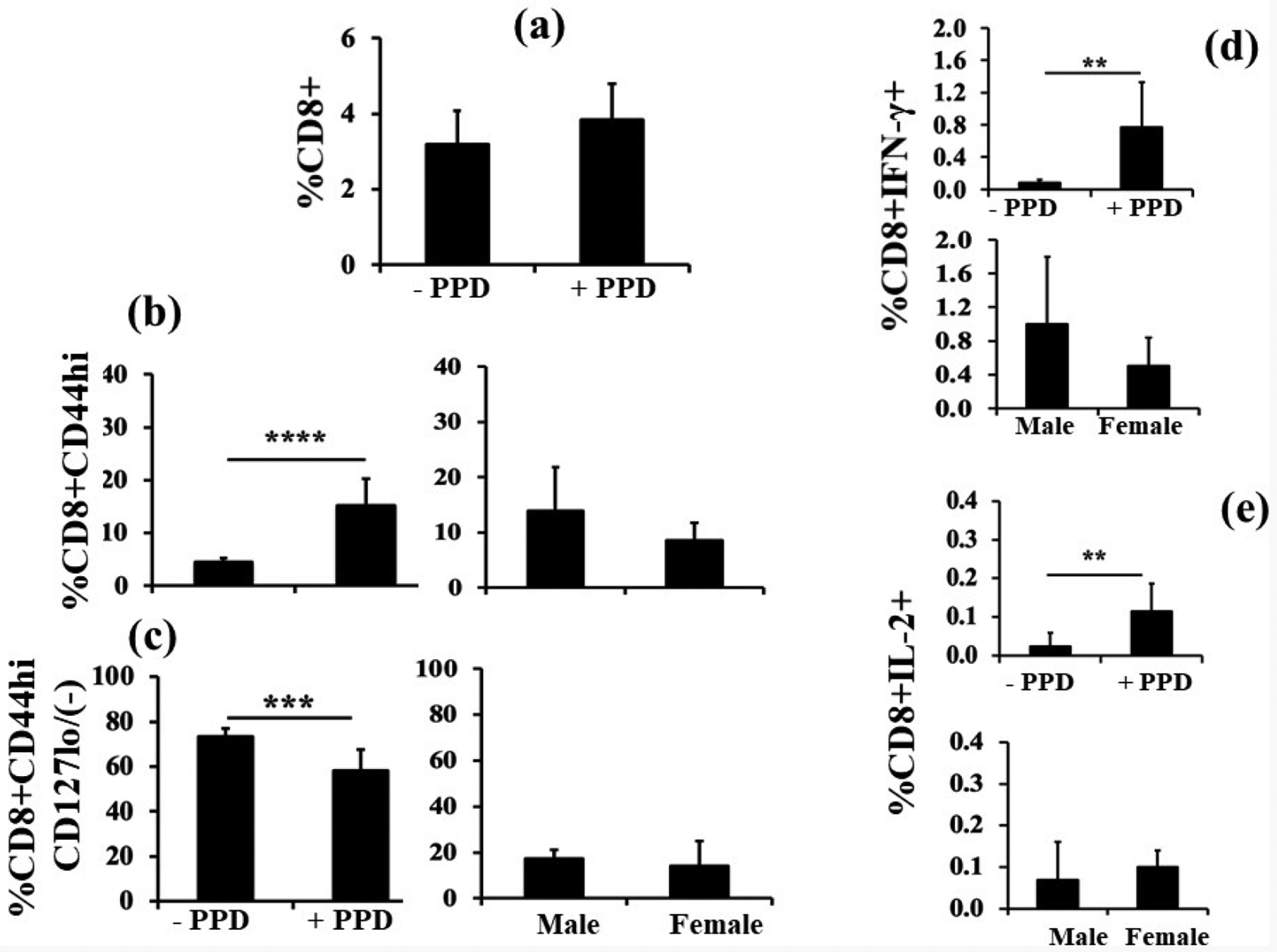
Adding LPS to the immunization protocol. Day 3 pups (n=8) were immunized with intra-peritoneal Mtb plus LPS and challenged with PPD on day 7 for 24 h before sacrifice. Isolated splenocytes were then stimulated with PPD for 16 – 18 h. Upon PPD stimulation no expansion of CD8^+^ T cells was evident (**3a**). Expansion of CD8^+^CD44hi T cells after PPD stimulation was evident (**3b**, left panel), however, there was no significant sex (male, n=3; female, n=5) difference (**3b**, right panel). Figure 3c shows CD8+CD44hiCD127(-/lo) T cells after PPD stimulation. This cell population contracted significantly (**3c**, left panel), but the contraction was not sex specific (**3c**, right panel). Figure 3d shows IFN-γ-producing CD8^+^ T cells after PPD stimulation. Stimulation with PPD significantly increased IFN-γ production by CD8^+^ T cells (**3d**, upper panel), but there was no significant difference between males and females after control subtraction (**3d**, bottom panel). Figure 3e shows IL-2 production in response to *ex vivo* PPD stimulation (**3e**, upper panel). After media control subtraction, there was no significant difference in IL-2 production between male and female pups (**3e**, lower panel). Data are expressed as means ± SD, and *p<0.05 was considered statistically significant. **p<0.01, ***p<0.001, ****p<0.0001.

To determine whether LPS in the environment influences the development of immunity in the neonatal period, mouse pups were immunized with Mtb plus LPS on neonatal day 3 and challenged with PPD on day 7 for 24 h. Neonates cannot tolerate LPS until day 3. Pup splenocytes were then stimulated with PPD *ex vivo*, as before. Under these circumstances, there was no significant expansion of total CD8^+^ T cells upon antigen stimulation (Figure 3a). By contrast, the CD8^+^CD44^hi^ T cell population increased significantly after PPD stimulation (Figure 3b, left panel) but just like in the pups without LPS, there was no significant difference between males and females (Figure 3b, right panel). As in the previous experiment, Figure 3c shows a contraction of the CD8^+^CD44^hi^CD127(−/lo) T cell population after PPD stimulation (left panel), but again no significant sex differences were evident (right panel). Figure 3d shows the percentage of splenic CD8^+^ T cells secreting IFN-γ in response to stimulation by PPD (upper panel) and, as seen previously, male *versus* female responses were not significantly different (lower panel). PPD stimulation also resulted in a significant increase in IL-2 production by CD8^+^ T cells (Figure 3e, upper panel) but, as before, there was no sex difference in IL-2 production by CD8^+^ T cells (Figure 3e, lower panel).

Thus, if analysis had stopped at this level, the conclusion would have been that there were no sex differences in the responses to either immunization protocol.

### Lack of PPD-specific bi-functional CD8^+^ T cells in male neonates.

**Figure 4.**
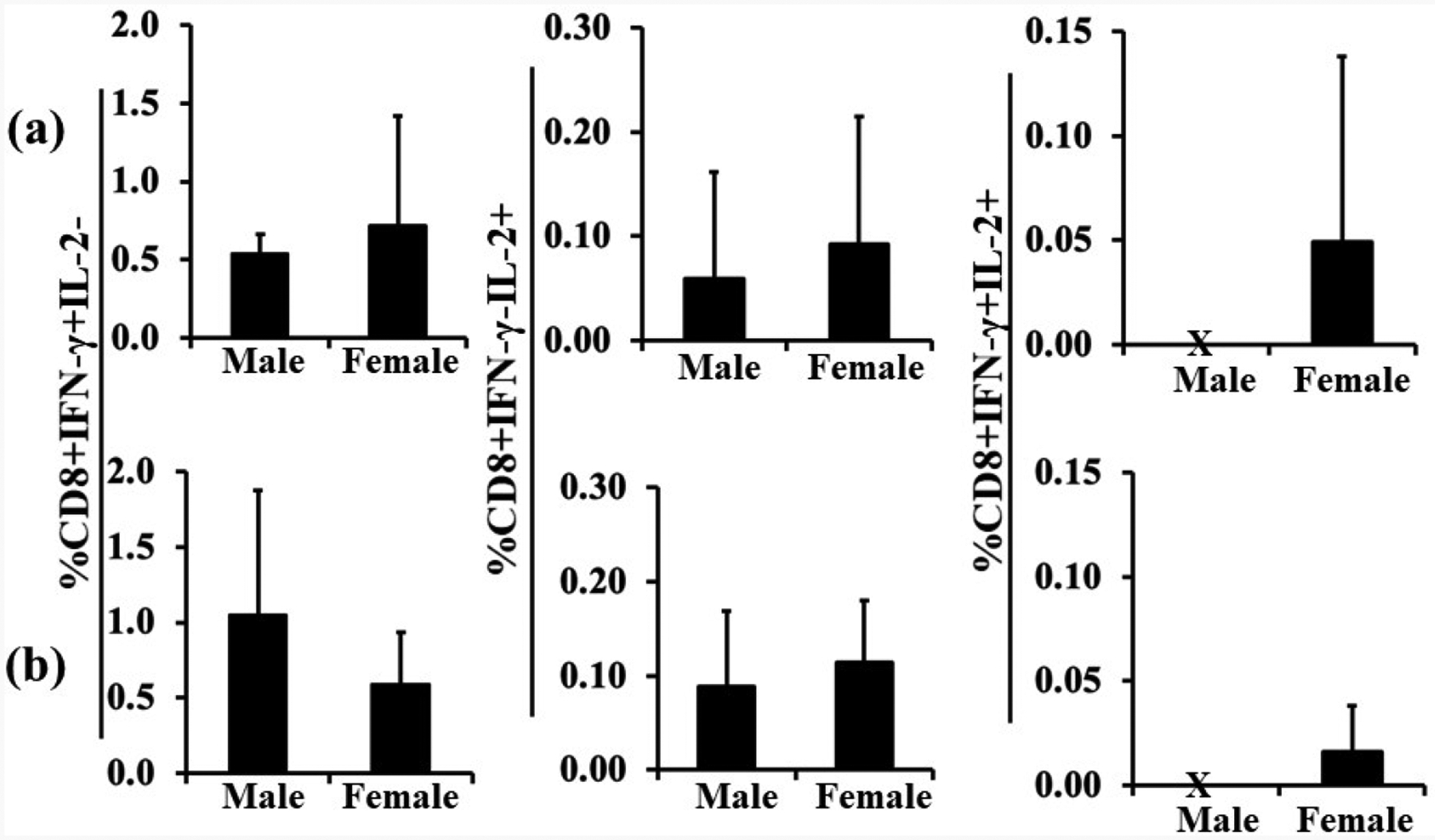
Boolean analyses of CD8+ T cell bi-functionality. After the same immunization and *ex vivo* stimulation protocol as for Figure 1a or inclusion of LPS in the protocol, as for Figure 3b, production of IL-2 or IFN-γ, or both, was separately assessed in males and females. In the two protocols, bi-functional CD8^+^ T cells positive for both IFN-γ and IL-2 (right panels in a and b) were detected in females (a, n=7, b, n=5) but not males (a, n=3; and b, n=3). Data are expressed as means ± SD.

Analysis of CD8^+^ T cells from pups in the groups originally immunized *in vivo* with either Mtb or Mtb plus LPS showed that both protocols induced production of IFN-γ in cells from all animals and that inclusion of LPS doubled the mean magnitude of the response in males (compare left panels in Figures 4a and 4b). IL-2 production was similar in magnitude with the two protocols but inclusion of LPS increased the proportion of both males and females responding (compare middle panels in Figures 4a and 4b). Interestingly, Boolean analysis for the production of both IFN-γ and IL-2 showed only cells from female animals were double positive regardless of group (see right hand panel in Figures 4a and 4b). Table 1 shows individual animals from both groups. Combining both groups (with and without LPS in the immunization) shows that 100% of animals responded by producing IFN-γ in CD8^+^ cells, that 67% of animals responded by producing IL-2 but that only females had cells that produced both IFN-γ and IL-2 (42% of females and 0% of males).

### T regulatory cells after *ex vivo* PPD-stimulation

**Figure 5.**
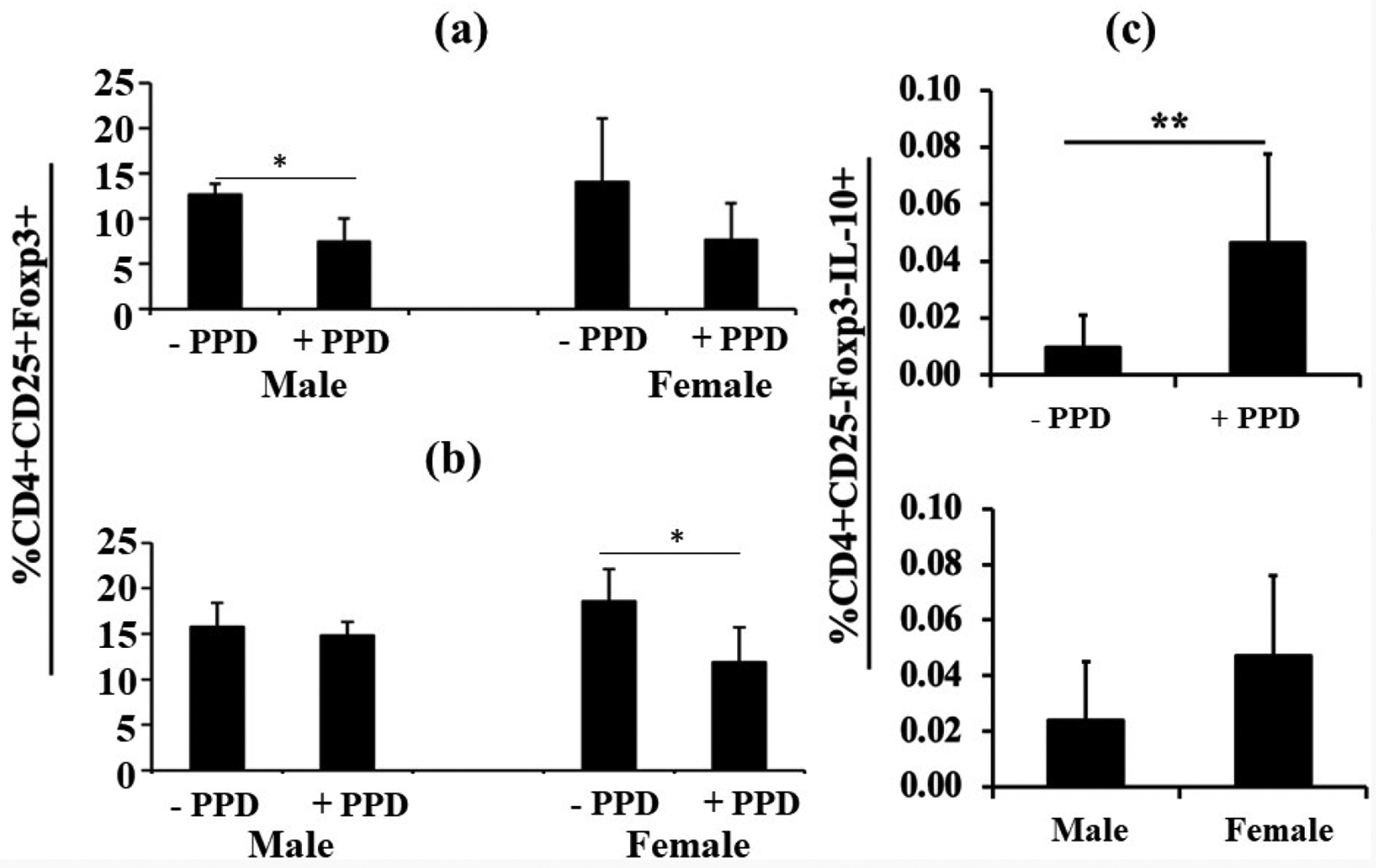
T regulatory cells after *ex vivo* PPD-stimulation. Again using the immunization protocol without (**5a**) or with LPS (**5b**), Treg and Tr1 Tregs were examined. Without LPS, there were small decreases in Tregs in both males (n=3) and females (n=7), although only the male response reached statistical significance (**5a**). With LPS, there was no change in males (n=3) and a decrease in females (n=7) (**5b**). Examination of IL-10-producing CD4^+^CD25^−^Foxp3^−^ Tr1 Tregs showed an increase in number in response to *ex vivo* PPD (**5c**, upper panel), but no difference between males (n=3) and females (n=7) (**5c**, lower panel. Data are expressed as means ± SD, and *p<0.05 was considered statistically significant. **p<0.01.

When splenocytes were obtained from pups from each group (Mtb only or Mtb plus LPS) and stimulated with PPD *ex vivo*, we noticed two distinct patterns with the two immunization protocols. With Mtb alone, there was a decrease in Tregs (CD4^+^CD25^+^Foxp3^+^) in male pups, and females also showed a decreasing trend, as one might predict (Figure 5a). However, in the LPS plus Mtb group, the percentage CD4^+^CD25^+^Foxp3^+^ cells decreased only in female pups, with no hint of change in males (Figure 5b).

There is a subset of CD4^+^ T cells, known as type 1 regulatory T (Tr1) cells, that are Foxp3 (−) and are induced under tolerogenic conditions in the periphery; a tolerogenic environment would be expected in newborn pups [16]. When Mtb immunized pup splenocytes were stimulated with PPD, there was a significant increase in the percentage of IL-10-secreting CD4^+^CD25^−^Foxp3^−^ Tr1 Treg cells (Figure 5c, upper panel), but no significant difference between males and females.

### Inclusion of LPS in the immunization protocol significantly increased IL-6 positive F4/80^+^CD86^+^ macrophages in female neonates as compared to their male littermates

**Figure 6.**
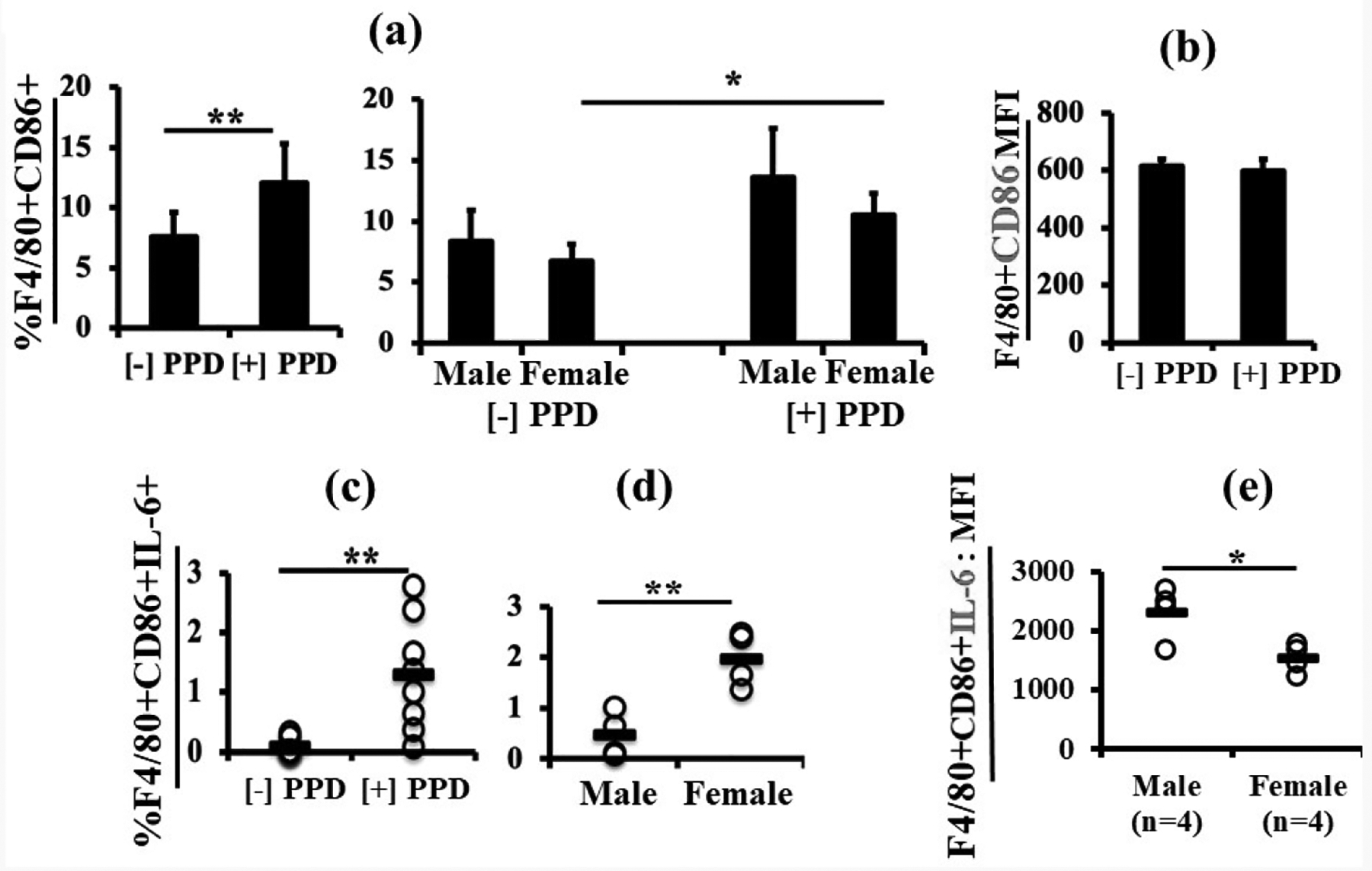
Inclusion of LPS in the immunization protocol significantly increased IL-6 positive F4/80^+^CD86^+^ macrophages in female neonates. Figure 6a shows expression of CD86 on F4/80^+^ macrophages, which increased significantly after PPD stimulation (**6a**, left panel), and when analyzed by sex, showed that only females (n=4) had significantly higher %CD86 expression (**6a**, right panel). However, the MFI of CD86 on F4/80^+^ cells did not change after *ex vivo* PPD stimulation (**6b**). Upon PPD stimulation the percentage of IL-6 secreting F4/80^+^CD86^+^ macrophages increased significantly (**6c**), and that in females (n=4) was significantly higher than males (n=4) (**6d**). Interestingly, the MFI of IL-6 was significantly higher in males (**6e**). Data are expressed as means ± SD, and *p<0.05 was considered statistically significant.**p<0.01.

*Ex vivo* PPD stimulation after *in vivo* immunization with Mtb plus LPS showed an increase in activated macrophages (F4/80^+^CD86^+^) in response to PPD that when further analyzed by sex was only significant in cells from female animals. (Figure 6a). Of the cells present, neither the mean fluorescence intensity (MFI) for F4/80 (not shown) nor CD86 differed between −PPD and +PPD (Figure 6b) or males and females (latter not shown). PPD stimulation also had a major positive effect on the percentage of F4/80^+^CD86^+^ macrophages also expressing interleukin-6 (IL-6) (Figure 6c). Interestingly, PPD-stimulated IL-6 expression by F4/80^+^CD86^+^ macrophages was significantly higher in females as compared to males (Figure 6d). However, the MFI of IL-6 in F4/80^+^CD86^+^ macrophages was significantly lower in females than males upon PPD stimulation (Figure 6e). Combined with the greater percentage IL-6 positive cells, this result suggests initiation of *ex vivo* production of IL-6 in female macrophages.

### PPD stimulation enhanced CD86 MFI and IL-6 expression in neonate CD11c+ dendritic cells

**Figure 7.**
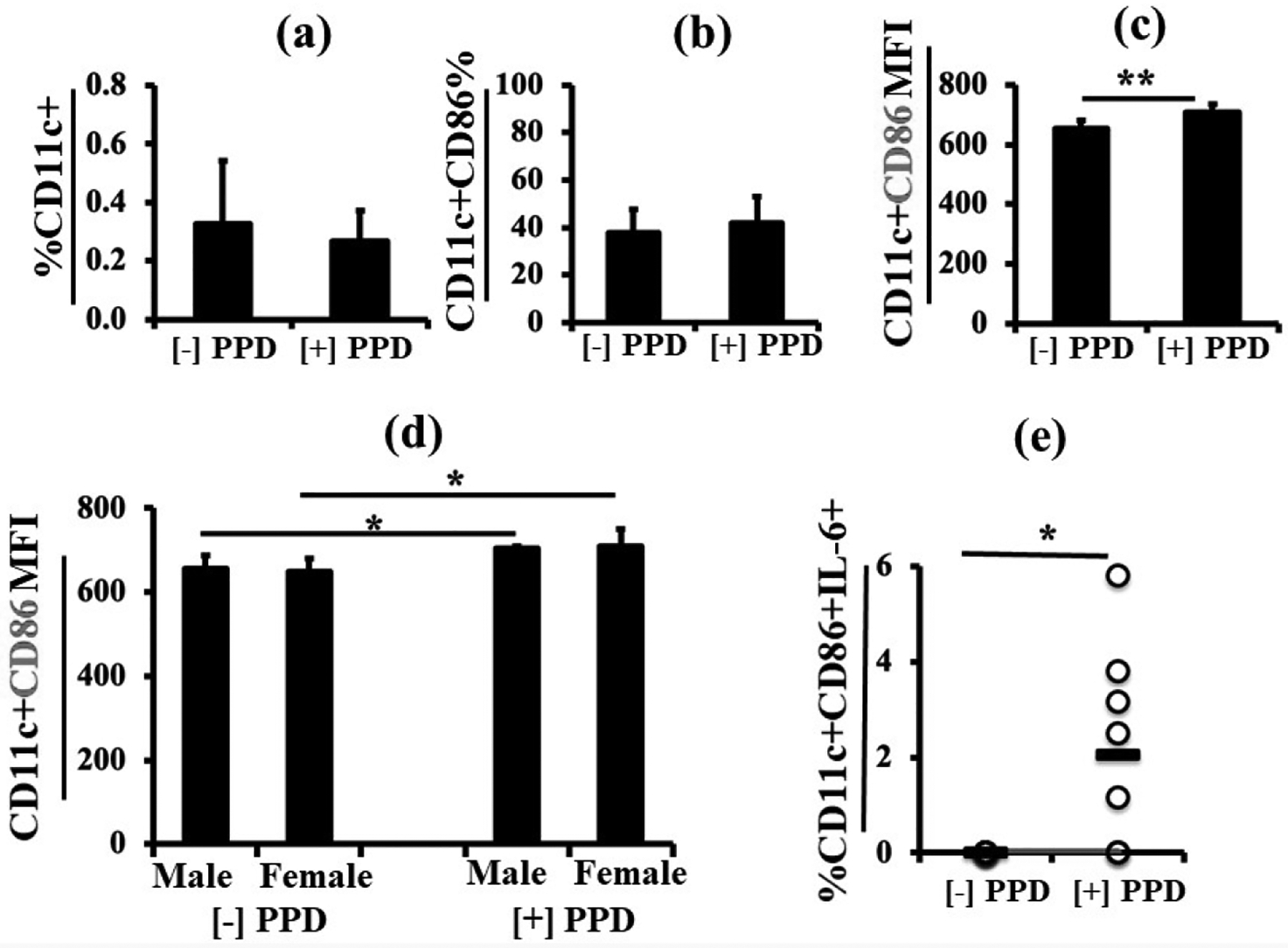
PPD stimulation enhanced CD86 MFI and IL-6 expression in neonate CD11c^+^ dendritic cells. Using the immunization protocol including LPS, showed that there was no increase in the percentage of CD11c^+^ dendritic cells after *ex vivo* PPD stimulation (**7a**), or in the %CD11c^+^CD86^+^ cells (**7b**). However, the MFI of CD86 increased (**7c**) and, in this case, increased in both males (n=4) and females (n=4) (**7d**). Importantly, PPD stimulation significantly increased IL-6 production by CD11c^+^CD86^+^ dendritic cells (**7e**). Data are expressed as means ± SD, and *p<0.05 was considered statistically significant. **p<0.01.

The percentage of CD11c^+^ dendritic cells in the Mtb Plus LPS immunized pup spleen remained unaltered after PPD stimulation (Figure 7a) as compared to control groups without PPD or when separated by sex (latter not shown).

Similarly, the percentage of CD11c^+^ cells also expressing CD86 did not increase after PPD *ex vivo* stimulation (Figure 7b). There was also no difference in the percentage of CD11c^+^CD86^+^ dendritic cells between male and female neonates with or without PPD stimulation (not shown). However, PPD stimulation significantly increased CD86 MFI in CD11c^+^ dendritic cells (Figure 7c), an increase that is apparent both in both males and females (Figure 7d). Thus, the magnitude of CD86 expression rather than the percentage of cells expressing CD86 in CD11c^+^ dendritic cells was increased in pup splenocytes after PPD stimulation. In addition, PPD stimulation significantly increased IL-6 production by CD11c^+^CD86^+^ cells as compared to un-stimulated controls (Figure 7e), although there was no significant difference between males and females (latter not shown).

## Discussion

Females are generally more immunocompetent than males [17,18]. In regard to TB, for example, the ratio of men to women suffering pulmonary tuberculosis is 7:3 [19]. Because many immune cells have receptors for gonadal steroids, analyses of sex differences in immune responses have largely focused on the reversible effects of gonadal steroids and their metabolites in adults [20]. Therefore, there is a generalized assumption that sex differences in immunity develop after puberty. However, using the FCG mouse model, we have previously determined that substantial sex differences manifest between days 1 and 7 after birth. Once developed, these sex differences are permanent and therefore organizational [6]. Experimental manipulation determined a role for both the male-specific [10] perinatal surge in testosterone and expression of the male-defining *Sry* gene that was independent of the testosterone effect [6]. There is no equivalent surge of either testosterone or estradiol in females [10]. The beauty of the FCG model is the ability to differentiate between effects due to chromosomal complement and those due to gonadal steroids, but a limitation is that the *Sry* gene is overexpressed [7]. Since our previous study determined that effects on CD8^+^ cell numbers were in large part the result of *Sry* expression [6] and CD8^+^ cell responses are a major component of TB immunity, it was important to conduct the current study in the background strain (C57BL/6J), which has normal expression of *Sry* and a normal perinatal surge in testosterone.

Immune responses of human infants are distinct and cannot be extrapolated from those of human adults [21,22]. This is also true for animal models where clear differences exist between neonates and older animals in response to intracellular pathogens, like Mtb. Thus, neonatal CD8^+^ cells preferentially give rise to short-lived effector and memory cells. Neonatal cells expand more rapidly, becoming terminally differentiated. Furthermore, it has been reported that there is an imbalance in effector and memory CD8^+^ T cells in neonates, with a shift toward more CD8^+^ T effector cells, an effect that is not due to a lack of responsiveness [23]. Also, Beijnen and van Haren [24] reported that neonates are less capable of creating immunological memory, which has implications for the development of adaptive immune responses after re-infection. In order to develop improved immunization protocols for human infants, it is therefore important to study neonatal immune responses and potential sex differences.

The TH1 cytokines, IFN-γ and TNF-α, are required for immunity against Mtb infection [25–27]. Both CD4^+^ [28–31] and CD8^+^ T cells [32,33,27] are important components of protection. However, there are reports of CD8^+^ T cell activation, predominantly the effector phenotype [34,35] with undetectable or low levels of IFN- γ, TNF-α and IL-2 production by CD8^+^ T cells [34,36,37]. Since our study was of neonates, it is also important to mention that neonatal CD8^+^ T cells are less cytotoxic due to lower expression of IFN-γ [38].

With both *in vivo* immunization protocols in the pups, PPD caused an *ex vivo* expansion of CD8^+^ cells, including the subset, CD8^+^CD44^hi^, thereby illustrating the ability of neonates to develop immunity to Mtb antigen. The significance of a reduction in the CD8^+^CD44^hi^CD127^lo/−^ population in response to *ex vivo* stimulation with PPD is unclear at present. However, Smith *et al*. [23] reported that neonatal CD8^+^ T cells preferentially give rise to short-lived effector cells, which may explain this reduction.

Analyzing CD8^+^ cell responses to *ex vivo* PPD in terms of total expansion, expansion of CD8^+^CD44^hi^ activated/effector cells, contraction of CD8^+^CD44^hi^CD127^lo/−^ and production of IFN-γ showed no significant sex differences, although based on individual animals, more female pups were capable of producing IFN-γ after PPD stimulation in both CD8^+^CD44^hi^CD127(−/lo) and CD8^+^CD44^hi^CD127^+^ T cell populations than their male littermates.

As expected, PPD-specific IL-2 production by CD8^+^ T cells was lower than IFN-γ production. Both neonate immunization groups produced IL-2 but, as in the case of IFN-γ, no sex difference in IL-2 production was evident.

Kollmann *et al*. [39] suggested that neonatal T cells are less likely to secrete multiple cytokines simultaneously, leading to less potent T cell responses [40]. One important finding in our study was a total lack of bi-functional (producing both IFN-γ and IL-2) CD8^+^ T cells in males, regardless of immunization protocol. Contrary to expectations [41], this lack of bifunctionality did not seem to correlate with a sex difference in Tregs or Tr1 tolerogenic cells. However, when LPS was part of the immunization protocol, only females showed a decrease in Tregs, suggesting an overall relatively pro-inflammatory environment in females.

LPS is an integral component of the outer membrane of Gram-negative bacteria [42] that enables bacteria to survive in harsh environments by creating a permeability barrier [43]. Such Gram-negative bacteria are a normal part of the environment for mouse and human neonates. Colonization is normal and is the rationale behind our use of co-immunization with LPS. One route of colonization is *via* maternal milk [11]. Gram-negative bacteria were isolated from maternal milk in 22.4% and 15% of mothers of preterm and term neonates, respectively. Moreover, genetically similar strains were present in maternal milk and the gut of 8.2% of preterm neonates. Given the activating properties of LPS on macrophages [13], we examined effects on macrophages in the neonates.

CD86 is a ligand for CD28 and CD152 (CTLA-4) and plays an essential role in regulating T cell immunity, balancing the activation and inhibition of T cell responses, respectively [44]. The ligand acts as an accessory molecule that plays an important role in T cell co-stimulatory interactions. Using CD86 as a measure of antigen-presentation, CD86-expressing F4/80^+^ macrophages increased significantly after PPD stimulation. F4/80^+^CD86^+^ macrophages also secreted more IL-6 after PPD stimulation, and the percentage of F4/80^+^CD86^+^IL-6^+^ macrophages was significantly higher in females as compared to males. This correlates well with reduced CD4^+^CD25^+^Foxp3^+^ Tregs observed in females but not males in the Mtb plus LPS immunized animals. Interestingly, when we compared IL-6 MFI between males and females, male cells had significantly higher MFI. Exactly how to interpret this result is a matter for debate at present and will require further investigation. For example, since Yang *et al*., [45] have shown that administered IL-6 suppressed neonatal immune responses, it is possible that for an optimal response, a neonate requires more cells producing a moderate amount of IL-6 (female) rather than fewer cells producing a larger quantity (male). Alternatively, the result may be indicative of rapid initiation of IL-6 production in the greater number of activated female macrophages in response to the overnight stimulation with PPD. In other words, female cells are primed to respond more rapidly but production of IL-6 per cell has not yet reached its peak.

*Ex vivo* PPD stimulation had no effect on the percentage of CD11c dendritic cells or the CD11c^+^CD86 population, but did increase the CD86 MFI and IL-6 positivity in both sexes, indicating activation, but the increases were not sex specific.

IL-6 is a pleiotropic cytokine that influences antigen-specific immune responses and the inflammatory response. IL-6 plays a very important role in regulating the balance between IL-17-producing Th17 cells and Tregs. IL-6 inhibits TGF-β-induced Treg differentiation and down-regulates Treg functional capabilities during an immune response. Th17 cells are key players in protection against bacterial infections, while Tregs function to restrain excessive effector T-cell responses [46]. A greater percentage of IL-6-secreting macrophages in female neonates indicates the potential to mount more effective immune responses to the PPD antigen.

Overall, our results demonstrate a more pro-inflammatory immune environment and more potent T cell responses in female neonates as compared to their male counterparts, suggesting more profound immune competency in females and one which is enhanced by exposure to LPS.

A limitation of our study is the physiological and immunological distance between mice and humans [47]. Compared to human infants, newborn mice are less developed, including immunologically [48] but Cooper [49] has opined that in TB vaccine development, the mouse model should be used as a tool to identify potential mechanisms of protection.

In summary, we have found evidence of substantial immune responses to Mtb in mouse neonates within the first week after birth, suggesting early immunization of human infants would produce some protection from TB. Female neonates exhibited a qualitatively superior response by producing bi-functional CD8^+^ cytotoxic T cells, which bi-functional cells are considered the most potent against infection; these were completely lacking in males. Female neonates also produced more pro-inflammatory, IL-6-secreting, antigen-presenting macrophages as compared to their male littermates. Since human males have two surges of pre-adolescent testosterone, one of which is intrauterine and the other of which is postnatal and peaks at about 1 month of age [50], extrapolation from mice to human infants suggests that immunization within the first few weeks of life may improve protection in males. The present study gave us, to the best of our knowledge, the first possible insight as to why male neonates are more susceptible to Mtb infection.

## Supplementary Material

JCI-25-225-Supplementary Figures

## Figures and Tables

**Table 1.** Lack of PPD-specific bi-functional CD8^+^ T cells in male neonates. Table 1 shows analysis of individual animals for the production of IFN-γ or IL-2, or both. Rows 2 and 3 show animals in which the immunization protocol did not include LPS, whereas rows 5 and 6 show results from the immunization protocol including LPS. Note that regardless of sex, 100% of the animals had CD8^+^ cells that produced IFN-γ in response to *ex vivo* PPD (second column). For IL-2 production in response to *ex vivo* PPD, note that LPS increased the number of animals that had CD8^+^ cells producing IL-2 and that a total (with and without LPS) of 67% of animals had cells that were positive for IL-2 (third column). The fourth column shows animals that had cells that were positive for both IFN-γ and IL-2. Regardless of immunization protocol, only females had bi-functional cells.

	IFN-γ[+] IL-2[−]	IFN-γ[−] IL-2[+]	IFN-γ[+] IL-2[+]
Male	3/3	1/3	0/3
Female	7/7	3/7	3/7
Male with LPS	3/3	3/3	0/3
Female with LPS	5/5	5/5	2/5
Male Total	6/6	4/6	0/6
Female Total	12/12	8/12	5/12
Percentage Male	100	67	0
Percentage Female	100	67	42
