# Supplementary figures and images for: The Ability of Neonatal Mice to Develop Immunity to Mycobacterium tuberculosis Shows Sex Differences, with Females Displaying Evidence of an Enhanced Immune Response
Source: J Cell Immunol. Author manuscript; Available in PMC 2025 Aug 16. (PMC12356363; doi:10.33696/immunology.7.225)

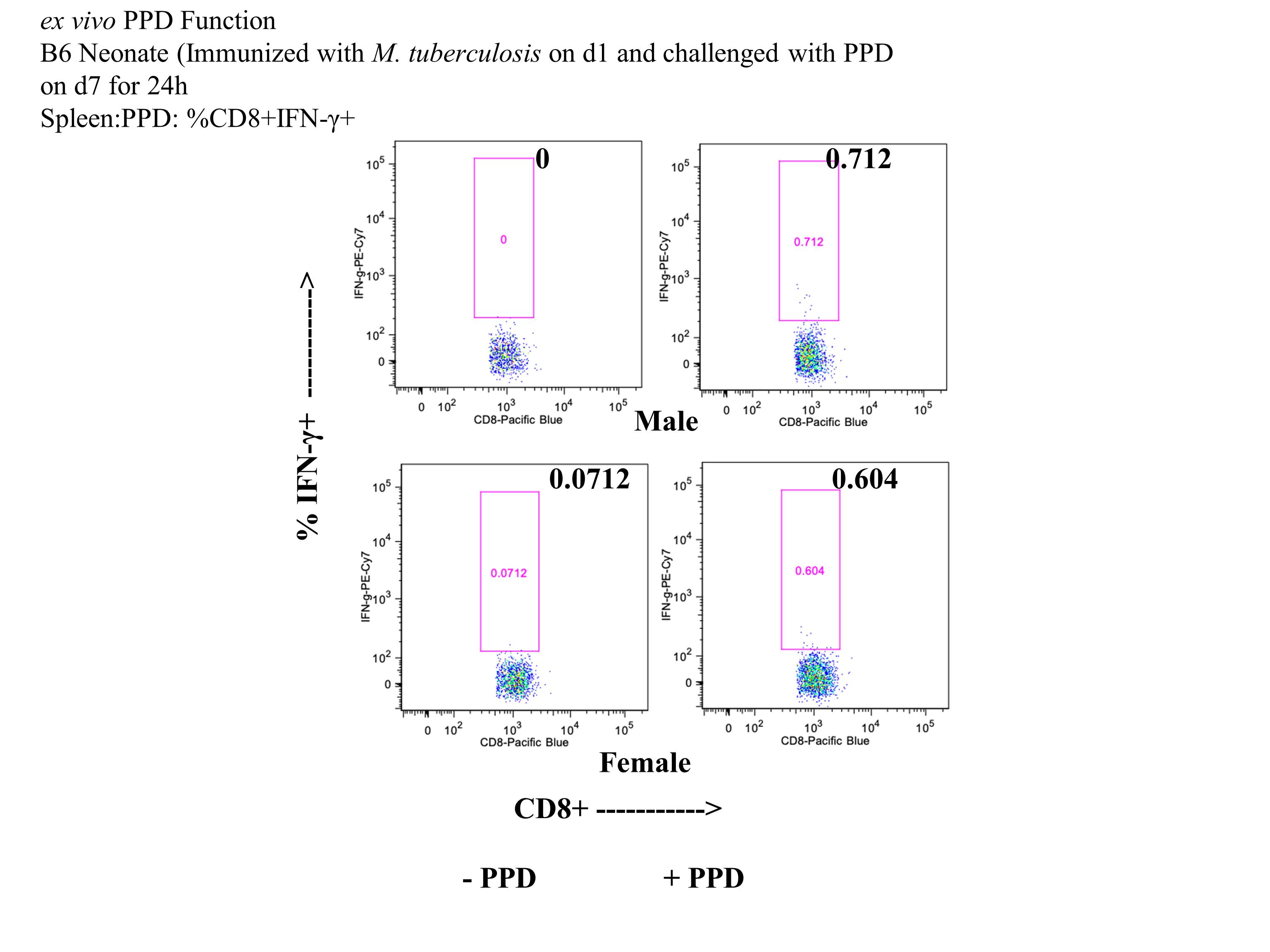

Supplement: JCI-25-225-Supplementary Figures [file NIHMS2101035-supplement-JCI-25-225-Supplementary_Figures.zip › JCI-25-225-Supplementary Figures/JCI-25-225-Supplementary-Figure 1.jpg]

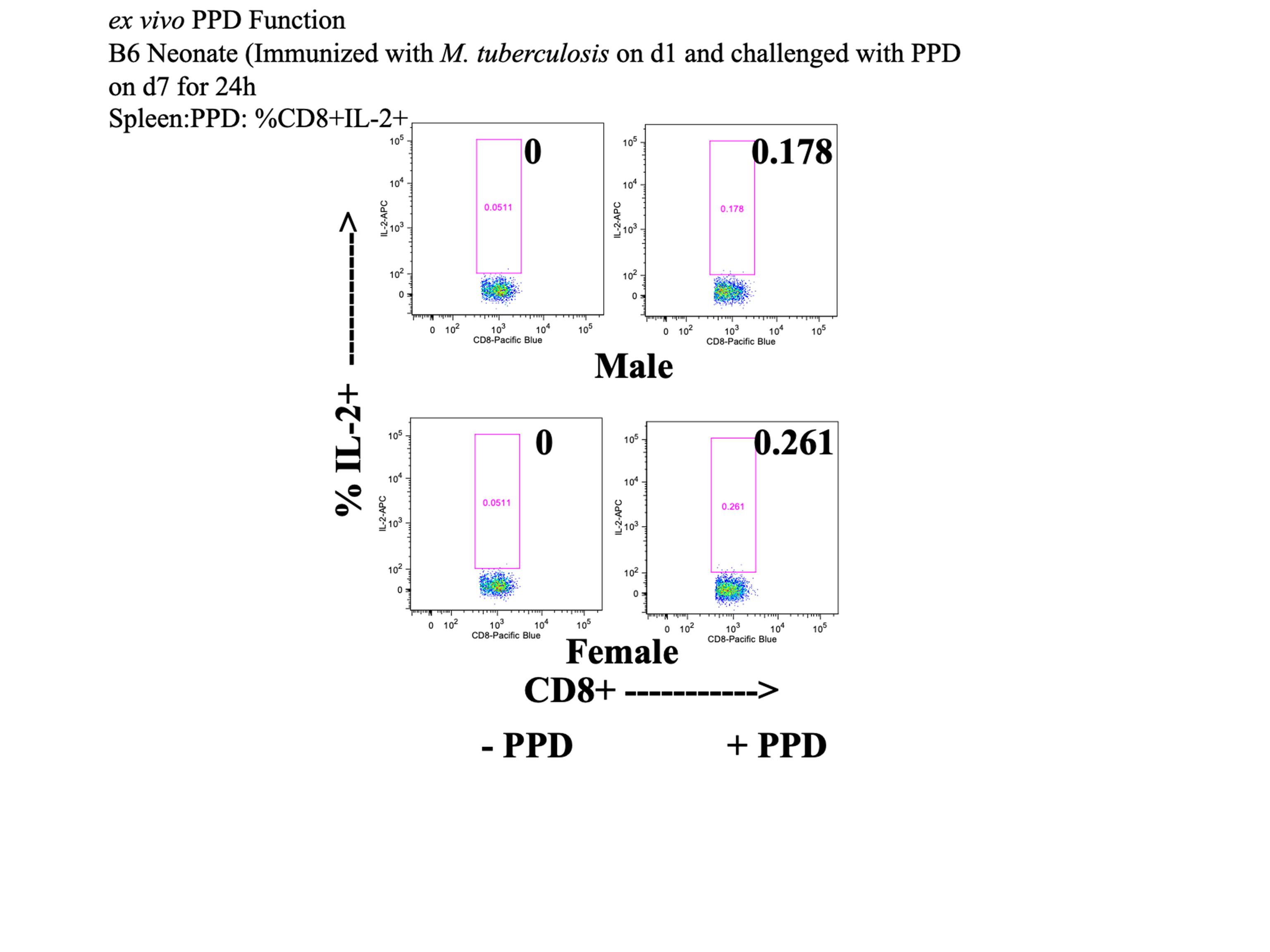

Supplement: JCI-25-225-Supplementary Figures [file NIHMS2101035-supplement-JCI-25-225-Supplementary_Figures.zip › JCI-25-225-Supplementary Figures/JCI-25-225-Supplementary-Figure 2.jpg]

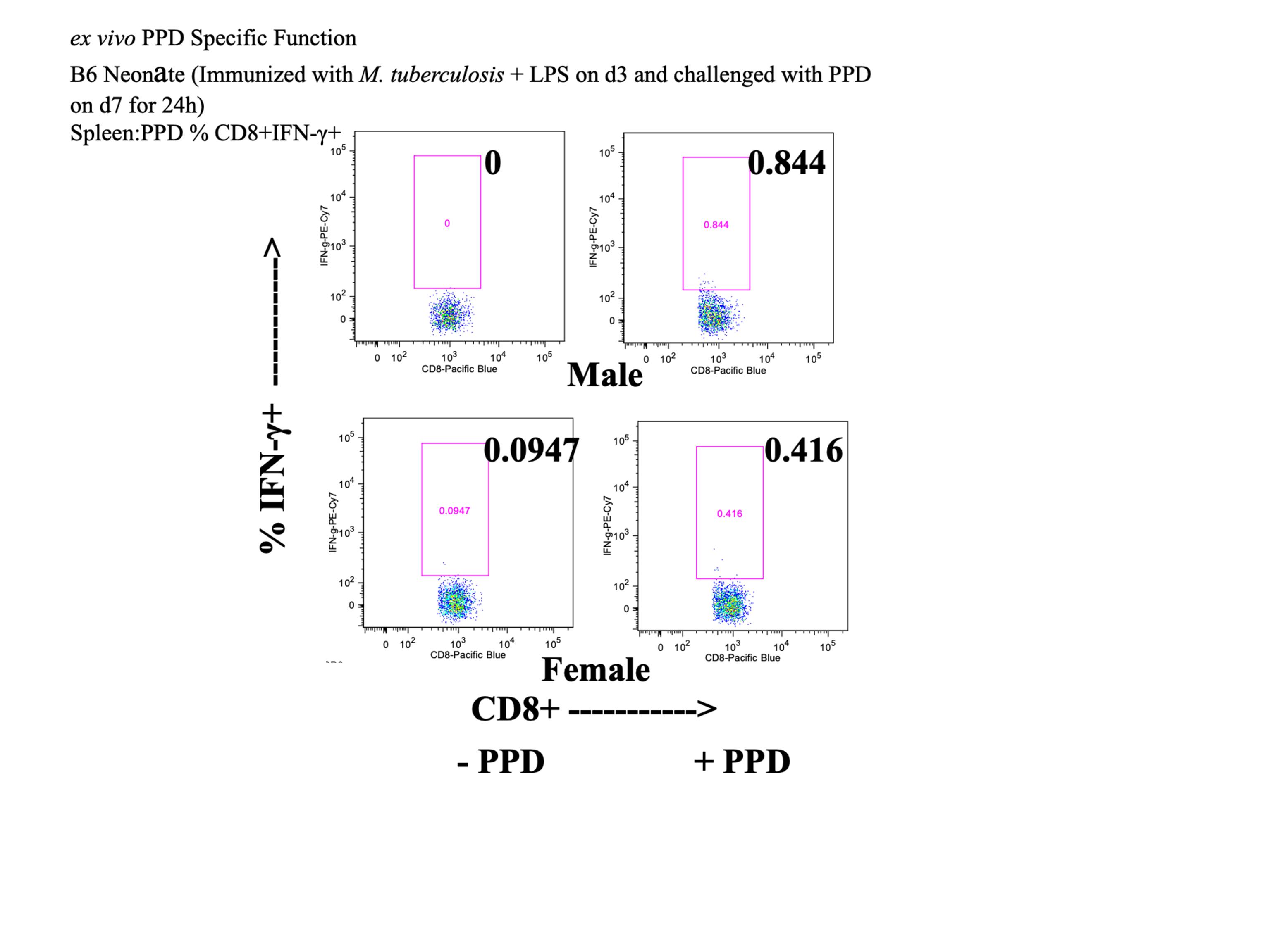

Supplement: JCI-25-225-Supplementary Figures [file NIHMS2101035-supplement-JCI-25-225-Supplementary_Figures.zip › JCI-25-225-Supplementary Figures/JCI-25-225-Supplementary-Figure 3.jpg]

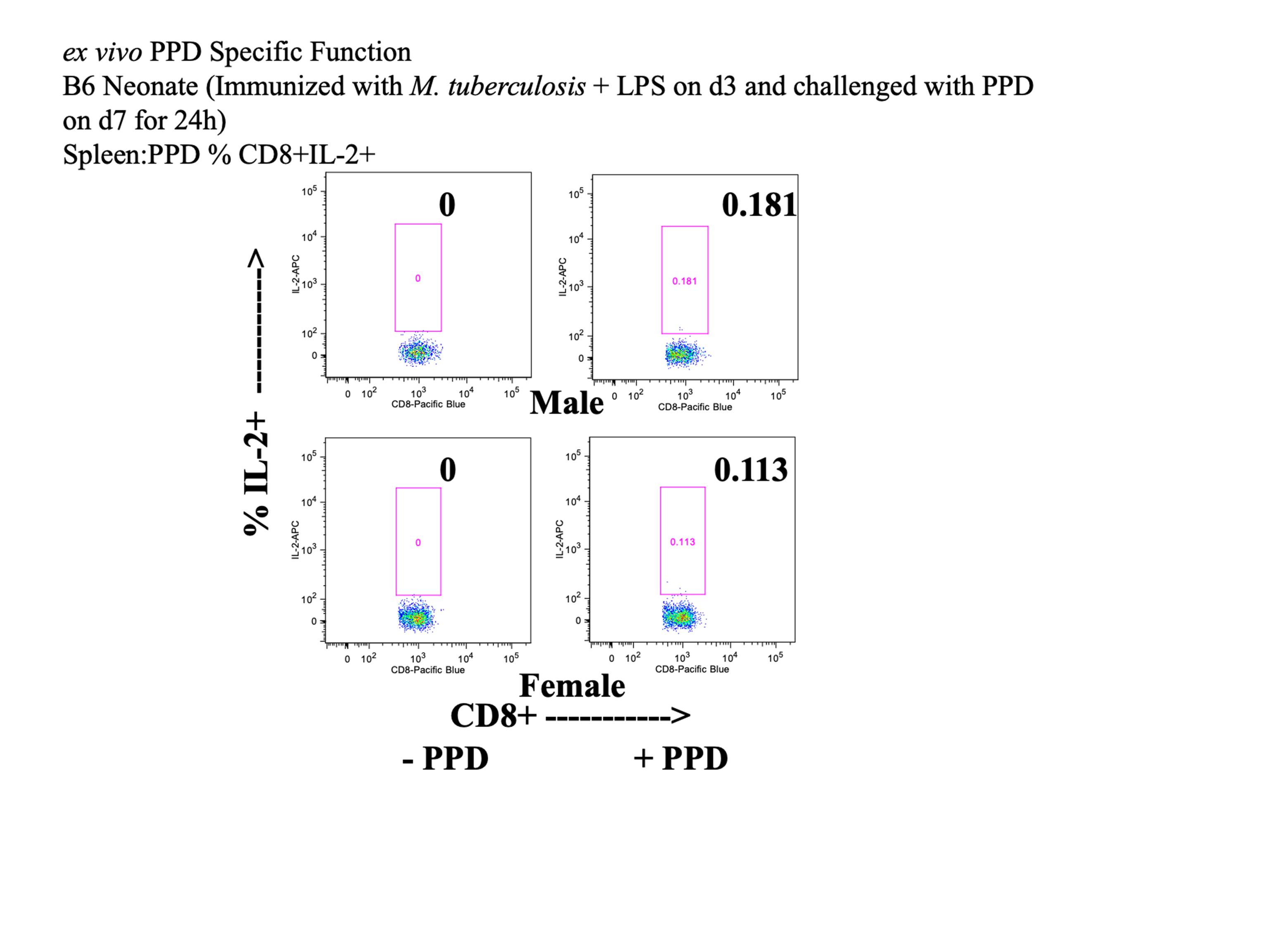

Supplement: JCI-25-225-Supplementary Figures [file NIHMS2101035-supplement-JCI-25-225-Supplementary_Figures.zip › JCI-25-225-Supplementary Figures/JCI-25-225-Supplementary-Figure 4.jpg]

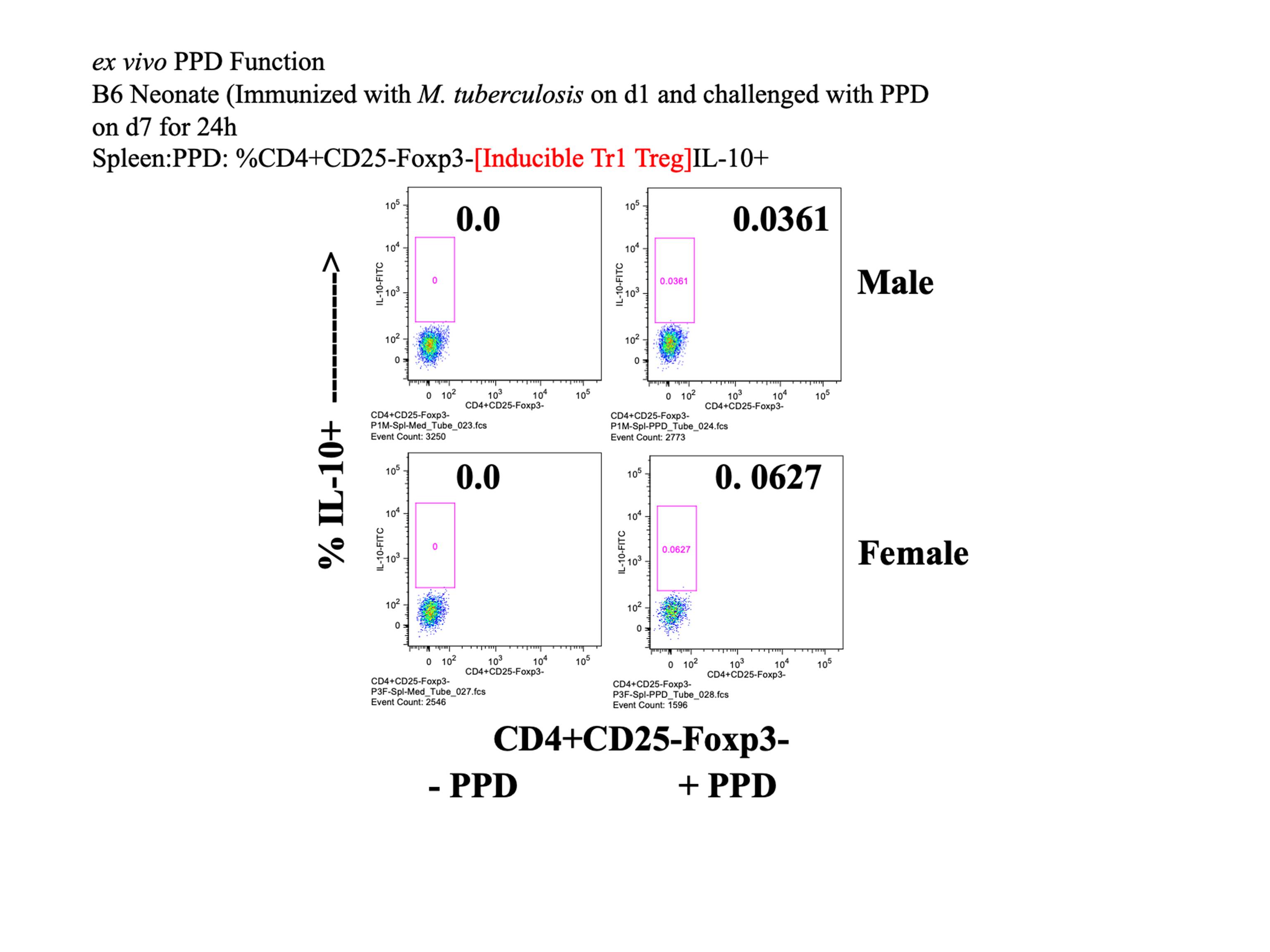

Supplement: JCI-25-225-Supplementary Figures [file NIHMS2101035-supplement-JCI-25-225-Supplementary_Figures.zip › JCI-25-225-Supplementary Figures/JCI-25-225-Supplementary-Figure 5.jpg]
